# Approaches to treating tuberculosis by encapsulating metal ions and anti-mycobacterial drugs utilizing nano- and microparticle technologies

**DOI:** 10.1042/ETLS20190154

**Published:** 2020-12-14

**Authors:** Khaled H. Alzahabi, Omar Usmani, Theoni K. Georgiou, Mary P. Ryan, Brian D. Robertson, Teresa D. Tetley, Alexandra E. Porter

**Affiliations:** 1 National Heart and Lung Institute, Imperial College London, London, U.K.; 2 Department of Materials and London Centre for Nanotechnology, Imperial College London, London, U.K.; 3 MRC Centre for Molecular Bacteriology and Infection, Imperial College London, London, U.K.

**Keywords:** antibiotics, inhalation, *Mycobacterium tuberculosis*, nanoparticles

## Abstract

Tuberculosis (TB) is caused by a bacterial infection that affects a number of human organs, primarily the lungs, but also the liver, spleen, and spine, causing key symptoms of fever, fatigue, and persistent cough, and if not treated properly, can be fatal. Every year, 10 million individuals become ill with active TB resulting with a mortality approximating 1.5 million. Current treatment guidelines recommend oral administration of a combination of first-line anti-TB drugs for at least 6 months. While efficacious under optimum conditions, ‘*Directly Observed Therapy Short-course’* (DOTS) is not without problems. The long treatment time and poor pharmacokinetics, alongside drug side effects lead to poor patient compliance and has accelerated the emergence of multi-drug resistant (MDR) organisms. All this, combined with the limited number of newly discovered TB drugs to treat MDR-TB and shorten standard therapy time, has highlighted the need for new targeted drug delivery systems. In this respect, there has been recent focus on micro- and nano-particle technologies to prepare organic or/and metal particles loaded with TB drugs to enhance their efficacy by targeted delivery *via* the inhaled route. In this review, we provide a brief overview of the current epidemiology of TB, and risk factors for progression of latent stage tuberculosis (LTBI) to the active TB. We identify current TB treatment regimens, newly discovered TB drugs, and identify studies that have used micro- or nano-particles technologies to design a reliable inhalation drug delivery system to treat TB more effectively.

## Introduction

TB, an airborne infectious disease caused by *Mycobacterium tuberculosis* (M.tb), is a major public health issue associated with a high rate of morbidity and mortality [1]. Pulmonary infection with M.tb. occurs when droplets (droplet nuclei, 1–5 microns) containing M.tb are released into the air by infected individuals and are inhaled and reach the deep lung including the respiratory alveolar units; the pathogen infects immune cells (alveolar macrophages) which surround the bacteria to contain the infection, leading to the formation of granulomas within the pulmonary tissues. Long-term immune-mediated containment of infection in such structures does not eradicate infection but can lead to latent TB where bacteria switch metabolism and can survive for years or decades in this state with no overt clinical signs (sub-clinical infection). Latent tuberculosis can reactivate and lead to active TB disease if the immune system is weakened [2], with risk factors including HIV infection, organ transplantation that involves immunosuppression, tumor necrosis factor alpha-blocker treatments, chronic renal failure, and hemodialysis [3,4].

The progression of Latent TB to active TB is often associated with the spreading of TB bacteria to extra-pulmonary sites including the spine, spleen, and liver resulting in serious symptoms of fatigue, weight loss, fever, and poor prognosis if the disease is not treated effectively [5]. Similarly, mismanagement of active TB can lead to multidrug-resistant TB (MDR-TB), where first-line anti-TB drugs such as rifampicin and isoniazid are no longer able to control and cure the disease and second and third-line treatments are required. MDR-TB accounts for 4.1% of new TB infections and 19.1% of previously infected patients [6].

M.tb has probably co-evolved in human populations over the last 15 000 or more years starting in East Africa and spreading to other continents as humans migrated out of Africa into northern Europe and along the rim of the Indian Ocean [7]. Tubercular lesions have been found in Egyptian mummies dating from 3400 BC [8], and Chinese and Indian documents from 2000 years ago describe skeletal changes (Pott's disease) associated with TB. In 1882, the M.tb organism was discovered by Robert Koch using a staining method he developed to differentiate the bacteria from the surrounding tissues which disclosed fine rod-like shaped structures within the tubercular granulomatous mass [9].

Currently, TB causes the largest number of deaths due to a single causative organism, with ∼1.5 million deaths per year, or one person dying every 20 seconds. Thus, a number of strategies have been instigated to reduce the incidence of infection and death in order to end the epidemic, for example, ensuring that all TB patients have access to affordable treatment. Correspondingly, the World Health Organization (WHO) End-TB strategy published in 2014, has targets to reduce TB death by 95% and new active TB cases by 90% by 2035, with intermediate milestones in 2020, 2025, and 2030 to assess global performance [10].

The continued large numbers of infections and deaths every year, with an increasing number of MDR- TB cases, illustrate the importance of developing more effective drug formulations to treat TB patients effectively. A significant problem is that TB drugs need to be taken orally at high doses over many months to achieve a cure, leading to toxicity, non-compliance, and drug resistance. Consequently, there is an urgent need to develop more effective drug delivery systems to directly target the affected tissues to reduce the dose of drugs, improve efficacy, and reduce side effects. One approach to treating pulmonary TB is to develop drug formulations that can be delivered directly to the granulomatous tissue in the lung by inhalation; for example, the use of engineered nano- and micrometer-sized particles as vectors to carry and deliver the drugs directly to the respiratory alveolar region, where these very small particles preferentially deposit once inhaled. Here we provide a brief background to the epidemiology and current treatment of TB followed by a detailed update of some of the new micro – and nano- technology approaches that are currently in development to treat TB and MDR-TB.

## Current epidemiology of tuberculosis

Every year, 10 million new active TB cases are reported globally — a prevalence that has changed little over many years [11]. In 2018, ∼1.5 million TB patients died, of whom 250 000 cases were diagnosed with positive human immunodeficiency virus/acquired immune deficiency syndrome (HIV/AIDS) [12]. The number of deaths has declined significantly, by 27% since 2000; however, this was accompanied by only a minor reduction, 1–5%, in TB cases with similar death rates observed in 2018 compared with 2017, and the same outcome was predicted for 2019 [13]. Although TB affects all age groups of men and women, the burden is higher in adult men (57% of confirmed TB cases in 2018), compared with adult women (32%), with children accounting for 11% [14]. Age appears to be an important influence on developing active TB; there is a higher tendency for infants and adolescents to develop active TB infection compared with those aged between 2–10 years or after 25 years of age [15]. Once LTBI occurs, there is a 5–15% chance of progression to active TB over a period of a few months to a few years [16].

**Figure 1.**
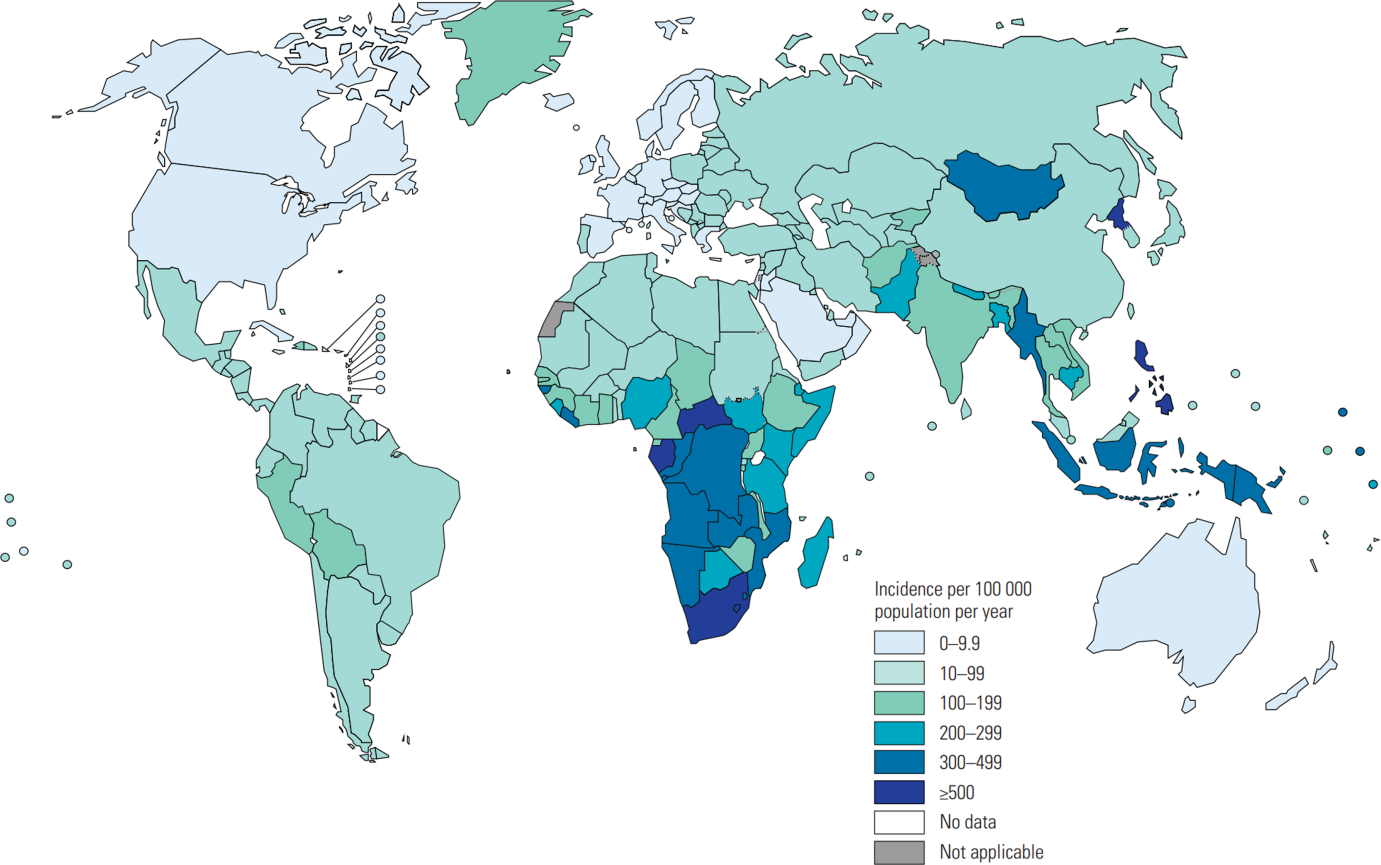
World Health Organization estimates of Mycobacterium tuberculosis (M.tb) infection incidence in 2019 [figure reproduced from 17].

Two-thirds of global TB cases in 2018 occurred in only eight countries namely: India, China, Indonesia, the Philippines, Pakistan, Nigeria, Bangladesh, and South Africa (in decreasing order of burden) and represent countries to target resources (Figure 1).

## Progression of LTBI to active TB

**Figure 2.**
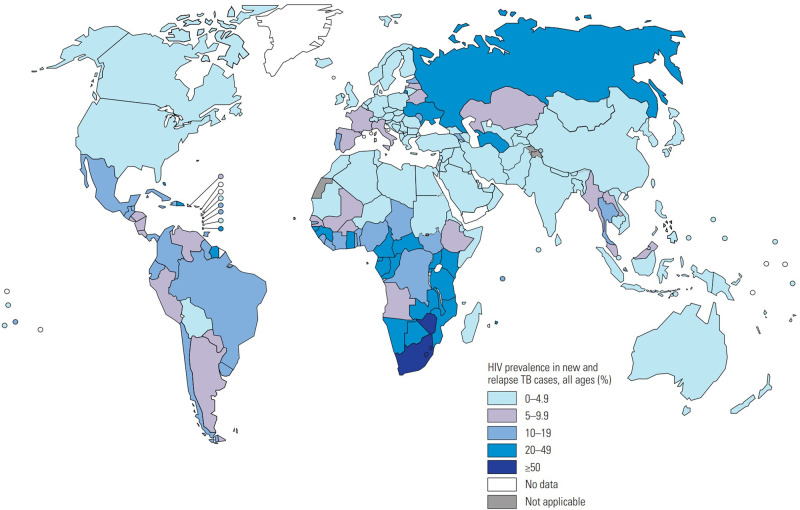
World Health Organization estimates of HIV prevalence in new and relapse TB cases in 2019 [figure reproduced from 17].

Many risk factors influence the progression of LTBI to active TB, including ongoing HIV infection (Figure 2), tobacco smoking, diabetes mellitus, malnutrition, air pollution, and consuming alcohol [3]. Importantly, the progression of LTBI to TB was reported to increase by 20-fold in those with HIV due to the depletion of CD4T-lymphocytes and other immune cells that control and contain LTBI [4]. The increased incidence of both diseases involves a decline in immunological responses which can lead to death if left untreated. The chance of people living with HIV (PLHIV) to get TB infection is 19 times higher than that for healthy people without HIV. In 2018, Africa had the highest-burden, 862 000patients, who were infected with both HIV and TB.

The chance of active TB development is 3-fold higher in diabetic patients and the death risk is 1.89 times greater compared with TB patients without diabetes [18]. The rate of diabetes (type 2 diabetes) is steadily increasing in poorer countries where TB is also prevalent, such as India, central and south America, and a few countries in Africa. Diabetes damages the adaptive and innate immune responses, again leading to a substantial increase in the survival and proliferation of M.tb. A recent study showed a remarkable number of M.tb organisms in diabetic mice with decreased production of IFN-γ and other cytokine mediators which resulted in weakened T-cell immunity. Furthermore, the recruitment of neutrophils was decreased in diabetic patients, enhancing the likelihood of progression to active TB [19]. Conversely, glucose intolerance and deterioration in glycaemic control are induced during TB which makes controlling diabetes more difficult [20].

Many studies have shown a strong link between tobacco smoking and active TB due to the negative effect of cigarette smoke on mucosal secretion defenses and the protective role of alveolar macrophages [21]. Nicotine markedly reduces the immune response; for example, the number of M.tb bacteria was increased in a model of mice exposed to tobacco smoke [22]. A similar effect was shown by consuming a large volume of alcohol, which also subdues the immune system and affects cytokine mediator production [23]. Similarly, air pollution substantially elevated the risk of active TB progression, and this effect was mainly associated with inhaling particles with a diameter less than 10 µm (PM10) and increasing levels of pollutant gases, nitrogen dioxide (NO2), nitrogen oxides (NOX), carbon monoxide (CO) and sulfur dioxide (SO2) [24]. The mechanism by which these pollutants increase the risk of TB was found to be multifactorial. For instance, diesel exhaust is found to affect macrophage function leading to reduced levels of tumor necrosis factor-alpha (TNF-α) and interferon-gamma (IFN-γ) which play important roles in controlling the activation of LTBI. Likewise, the interleukin-10 (IL-10) level was increased for mice exposed to CO particles resulting in a higher risk of progression to active TB [25]. Furthermore, the risk of developing active TB was shown to increase substantially, between 6–10-fold, with malnutrition [26]. This is exacerbated in those with TB who exhibit reduced appetite, malabsorption of nutrients, and wasting [27].

In addition to these risk factors, genetic variation in both humans and the different strains of the M.tb pathogens can impact on the prevalence, morbidity, and mortality of TB. Genetic variations in humans also affect the progression of LTBI into active TB [28]. This is a complex area and regardless of the underlying predispositions to acquire TB and progress to active TB, effective targeted treatment that will rapidly kill the organisms and reduce transmission, and prevent the development of MDR-TB is a desirable outcome.

## Current TB treatment guideline and vaccination

Thus, the main aims of TB-therapy are to cure patients effectively, reduce transmission of infection to other people, and prevent the occurrence of MDR-TB [29]. The WHO recommended ‘Directly Observed Therapy Short-course’ (DOTS) treatment for drug-sensitive TB consists of a 2-month intensive phase with 4 drugs (Rifampin (RIF), Isoniazid (INH), Pyrazinamide (PZA), and Ethambutol (EMB) taken daily, followed by a continuation phase of RIF plus INH daily for 4 months. Patients must be observed taking their medication to ensure compliance and under these circumstances, cure rates can exceed 85%. There are a number of variations of DOTS depending on factors such as co-infection with HIV or other co-morbidities, and MDR-TB requires longer treatment with more toxic drugs [30,31].

Bacillus Calmette-Guérin (BCG) is the only licensed vaccine available for TB and is most effective when given to neonates in high burden countries and provides protection against disseminated forms of tuberculosis. It works less well in older children, especially those in high burden countries, for reasons that are not fully understood [32].

## Promising new TB drugs and vaccines

The lengthy, complex drug treatment regimen, and toxic side effects — which lead to patient non- compliance — are major obstacles to successful TB therapy and contribute to the development of MDR- TB. Thus, new drugs and targeted drug therapy formulations are urgently required to overcome the current challenge of eradicating TB as a global health problem. This means that new treatments need to be shorter, simpler, safer, and affordable, with fewer adverse drug interactions [33]. There are over 20 promising anti-mycobacterial agents in various stages of clinical development, or recently licensed [11,34]. These are either new chemical entities or derivatives of existing compounds, and include drugs with novel targets, such as Bedaquiline, which inhibits the mycobacterial ATP synthase, and drugs with known targets such as Sutezolid and Delpazolid which both inhibit protein synthesis. However, these agents fall into around only 10 functional classes, targeting, for example, translation, lipid transport, lipid biosynthesis and catabolism, ATP synthase, meaning that their long-term value may be undermined by the potential for cross-resistance. Moreover, many drugs that have been approved for other conditions are being tested in combination with current multi-drug therapy, including higher doses of more potent, longer-lasting Rifamycins, and fluoroquinolones such as moxifloxacin.

Similarly, ∼20 novel vaccines are in clinical trials at various stages and include modified versions of the current BCG vaccine, protein subunit vaccines delivered with adjuvant, recombinant viral vectors expressing one or more mycobacterial antigens, and live attenuated M.tb [35]. In the one hundred years since the original BCG was developed, nothing better has emerged. The continued quest for new vaccines in the face of uncontrolled TB reflects our lack of insight to the exact immunological mechanisms involved hence the inability to effectively contain, or protect people from, the disease.

## Micro- and nano- technology for treatment of TB

The availability of new anti-TB drugs may not be sufficient to combat MDR-TB and eradicate TB at the rate required to meet the WHO END-TB plan. Finding alternative approaches to overcome the poor pharmacokinetics and pharmacodynamics of currently used oral TB drugs could advance the treatment of this disease [36]. First-line TB treatments have limited solubility in water which reduces their absorption from the gastrointestinal tract leading to low bioavailability [37], even when high doses are taken. They also have short half-lives and are eliminated rapidly from the body [38]. Consequently, oral or even intravenous administration of these drugs may deliver insufficient therapeutic dose to the affected organ and particularly the lungs [39]. The interest in utilizing unique nanotechnology approaches for the treatment of TB has increased substantially over recent years, particularly for targeted inhalation therapy, due to the potential advantages this has over conventional treatments. This relates to the ability of the inhalation system to deliver the drug to the site of infection, accelerate the onset of treatment using potentially less dose of drugs and mimicking the way that M.tb harnesses the host defence and other mechanisms to infect lung through M.tb-contaminated aerosol. However, a challenge for inhalation therapy is to produce an optimal biopharmaceutical formulation that is physically and chemically stable and efficiently and reproducibly delivered to the lungs at the requisite doses needed to achieve clinical efficacy, with favourable pharmacokinetic profiles.

## Nano- and micro- particles for targeted therapy of TB

Several studies have investigated the use of nano- or microparticles (NP and MPs, respectively) loaded with traditional TB drugs as inhalation therapy for TB. Formulations include the use of different nano- or micro-carriers, including nanoemulsions, liposomes, nanosuspensions, and polymers, to encapsulate and deliver antitubercular drug(s) to the infection site. Nanoemulsions are thermodynamically stable oil-in-water dispersions with droplet size ranging between 10 and 100 nm. Particle dispersion can be stabilized by various surfactants and cosurfactants, producing large interfacial areas, and a stable dispersion and stability for inhalation therapy. For example, a nanoemulsion encapsulating Rifampicin showed an extended drug-release profile of up to 2 h, with a drug entrapment efficiency of 100% and osmotic pressure that compares with blood and is suitable for IV delivery [40]. Liposomes are made up of spherical vesicles composed of a phospholipid bilayer; their unique structure gives them the ability to deliver hydrophobic, amphipathic, and hydrophilic medicines. Nanosuspensions are biphasic systems consisting of pure drug particles in aqueous vehicles. The vehicles are generally composed of either surfactants or polymers that stabilize the nanosized particles. These nanosuspensions are used to increase the solubility and bioavailability of drugs with strong intermolecular interactions that cause low solubility in aqueous and oily media. In a nanosuspension form, Rifampicin's solubility was increased 50 times compared with the free drug and the cytotoxicity on epithelial lung cells was reduced. In one study, Rifampicin nanoparticles were prepared using a microemulsion-based technique, cetyl palmitate, and Tween 80 [41]. This resulted in spherical Rifampicin nanoparticles with a size of 100 nm, low negative zeta potential, and encapsulation efficiency of 82% were produced. Rifampicin was released with a sustained drug release profile for 72 h showing an improved antibacterial effect against *M. fortuitum*, being effective at one eighth the concentration of Rifampicin alone.

Table 1 lists other recent approaches to utilizing these micro- and nanotechnologies to design efficient antitubercular lipid-based therapies. The results given in the table show that encapsulating a single TB drug within nano- or micro-particles enhanced the release profile, improved the antitubercular efficacy, and produced reliable drug delivery systems that can be administered by several routes, such as oral, i.v. or inhalation. Importantly, dual loading of different TB drugs within nanoparticles showed better antitubercular results compared with the mixture of free drugs. These results are promising for future research aimed at designing nano- or micro-particles containing multiple TB drugs. Interestingly, nanotechnology could also play an important role in vaccination, or enhance the effects of vaccines used to treat or prevent TB. For example, vaccination using a plasmid DNA vector encoding the antigen 85A (Ag85A) of M.tb was facilitated when adsorbed to poly(D,L-lactide-*co*-glycolide) (PLGA) microparticles and delivered intramuscularly. Thus, similar protection to that seen with BCG was observed with the PLGA-adsorbed DNA encoding Ag85A, which in turn was 100 times more effective than naked DNA-encoding Ag85A, when assessed in an aerosol challenged mouse model of M.tb [42,43].

**Table 1:** Recent studies utilizing micro/nanotechnology in TB drugs since 2015

	Aim	TB drug	Excipients	Dosage form	Method	Results
1 [64]	Design a pulmonary drug delivery of RIF	RIF	Soluplus	Micelles	A solvent-diffusion technique was employed to prepare the nanocarriers. Characterization: Size, morphology, *in vitro* release, stability, and cytotoxicity.	The solubility of RIF increased significantly when micelles (with a diameter of 107 nm) were formulated. The *in vitro* TB efficacy of the RIF-micelles was greater than the free drug at 24 h *in vitro*.
2 [65]	Encapsulation of many first-line TB drugs in one dosage form	RIF, NIH, PZA	Ethyl oleate (oil phase), Brij 96 (surfactant), butanol (Co- surfactant), and distilled water	Microe mulsion	A microemulsion consisting of an oil, surfactant, cosurfactant, and double-distilled water with constant surfactant-cosurfactant mass ratio was formulated. Characterization: Size, viscosity, loading efficiency, drug release, toxicity to Vero cells, and efficacy against several positive and negative gram bacteria.	The viscosity of the microemulsion did not change when the drugs were added and RIF (the most hydrophobic drug) was encapsulated within the core of oil droplet. INH was adsorbed to the aqueous side of the water-oil interface, and PZA (the most hydrophilic drug) was mainly dissolved in the aqueous bulk. The system was stable for 20 days at room temperature. The release profile followed a diffusional profile for INH and PZA, with analogous release for RIF. Toxicity was dependent on the concentrations but higher with ethyl oleate. The maximum antibacterial effect was observed for the RIF microemulsion and the RIF, PZA combination.
3 [66]	Compare the inhalation and oral routes of delivery of RIF microparticles	RIF	Dichloromethane	MP	MPs were prepared by the spray-drying method. Characterization: Size, aerodynamic diameter, *in vitro* release in simulated phagosomal and gastric fluids, *in vivo* deposition of the drugs.	Particles were produced with a median particle size of 3.6 ± 0.77 µm, a mass median aerodynamic diameter of 2.5 ± 0.061 µm, and emitted dose of 58.68 ± 0.84%. Full release of RIF was complete in 2 h in gastric fluid but only 53.35% was dissolved in phagosomal fluids. A high concentration of RIF was found in alveolar macrophages. No signs of hepatotoxicity were measured.
4 [62]	Prepare an inhaled delivery of RPN	RPN	PLGA	MP	MPs prepared by spray drying/oil-in- water (O/W) single emulsion solvent evaporation. Characterization: Morphology, size, thermal analysis, drug loading, *in vitro* dissolution, cytotoxicity, and macrophage uptake using THP-1 monocyte-derived macrophages.	Spherical MPs were prepared with a volume median size of 2 µm. 90% of the PLGA MPs were encapsulated by macrophages and the MPs were not toxic to the cells. The monomer molecular weight and composition did not influence the aerosol's performance and toxicity but increasing the lactide concentration enhanced uptake by macrophages.
5 [67]	The development of effective and safe nanotechnology-based methods	BDQ	Tween® 20, EtOH,Span® 85 (sorbitanetrioleate), Oleic acid, and chitosan	Nano capsule	NPs were prepared by blending lipid and aqueous phases using an ultrasonic process to provide stable nanodroplets.Characterization: Encapsulation efficiency, drug loading*, in vitro* efficacy against Mycobacterium tuberculosis, and cytotoxicity on animal cells.	Nanocapsules successfully encapsulated the drug. The antibacterial efficacy was increased significantly, with rapid interaction with TB bacteria. No cytotoxicity was shown towards animal cells for any of the therapeutic concentrations of the drug.
6 [68]	Achieve synergistic treatment using nanoparticles	INH, MX	Chloroacetyl chloride (CAC) & succinyl chloride (SCL) to modify the drugs and PLGA for encapsulation	NP	NPs were prepared by a single emulsion reaction after linking the drugs together through nucleophilic substitution reaction. Characterization: Compatibility, particle size, size distribution, Zeta potential, drug entrapment efficiency, drug release, and accelerated stability studies.	An enhanced effect of the two drugs was achieved, when they were delivered inside the NPs and the NP formulation achieved better antibacterial activity than the free mixture of the drugs.
7 [69]	Deliver ETH and its booster effectively to the lung	ETH, BDM 41906	Poly (lactic acid) (PLA) and PLGA	NP	Different forms of nanoencapsulation were prepared. Nanoprecipitation of ETH with PLGA and PLA was performed using DMSO. A nano- emulsion of ETH was prepared using MeOH and dichloromethane (DCM). ETH and its booster were encapsulated without using solvents. Characterization: Shape, size, zeta potential, drug loading and their effect on mouse macrophage RAW 264.7 infected by the M. tuberculosis H37Rv strain.	300 nm NPs were prepared with the solvent, with a loading efficiency of 77% Without the use of the solvent, 10 nm NPs were produced. The NPs were delivered to the lungs using a Microsprayer® which gave a decrease by 3-log of pulmonary mycobacterial count after 6 administrations, suggesting that a combination of ETH and its booster increased the potency of the individual drugs.
8 [70]	Enhance the bioavailability of the drug	RIF	Compritol 888 ATO,Span® 80 and stearylamine	NP	NPs were prepared by an O/W microemulsion method followed by a high-pressure homogenization technique.Characterization: Size, zeta potential, entrapment efficiency, drug loading, morphology, *in vitro* release, and stability study for 6 months at 8 °C, 30 ± 2°C/65 ± 5% RH, and 40 ± 2°C/75 ± 5% RH.	NPs with a diameter of 456 ± 11 nm and encapsulation efficiency of 84.12 ± 2.78%, were stable in different simulated gastrointestinal tract media. The drug was released in biphasic profile with 90% of the drug was released within 120 min and the best fitting model was Weibull. An accelerated stability test did not alter the physical stability of the NPs.
9 [71]	Design a controlled release of IV delivery	LZ	PLGA 752 H	NP	Nanoparticles were prepared by a solvent evaporation technique using high speed homogenization. Characterization: Size, zeta potential, encapsulation efficiency, antibacterial efficacy, *in vitro* dissolution test using lung simulated fluid, and *in vitro* deposition in the lung using an Anderson Cascade Impactor.	NPs were prepared with a size <300 nm with a correlation between the size and homogenization speed and time. The NPs had an encapsulation efficiency up to 85.33%. These NPs showed a sustained release up to 2 h. The deposition studies showed the particles have potential to reach the alveolar unit *in vivo*.
10 [72]	Improve TB treatment by designing RIF nanoparticles	RIF	PrecirolR ATO 5polysorbate 60 and miglyol-812	NP	High shear homogenization and ultrasonication techniques were used to prepare the particles and they were coated with mannose. Characterization: Size, zeta potential, drug loading, morphology, toxicity on Bone marrow-derived macrophages, drug efficacy on the Mycobacterium avium strain 2447.	Spherical NPs with a mean size of 315 nm, and encapsulation efficiency of 95% were prepared. Efficient uptake of mannosylated NPs by bone marrow-derived macrophages was observed and improved antibacterial efficacy was observed for RIF mannosylated nanoparticles compared with the free RIF.
11 [73]	Design a pulmonary delivery of ETM to the lungs	ETM	Compritol and Tween 80	NP	Hot homogenization and ultrasonication methods were used to prepare the NPs and the dry powder inhaler was prepared by spray drying. Characterization: Flowability, deposition, encapsulation, size, and toxicity on a A549 cell line.	An encapsulation efficiency of 98% was achieved with a NP size of< 100 nm. Release of EMT from the nanoparticles was less than the free drug with 34% after 8 h. No significant toxicity was observed for any of the NP formulations compared with the free drugs.
12 [74]	Study the effect of human serum albumin as a carrier to deliver TB drug	BTZ	Albumin	NP	NPs were prepared by a modified desolvation method. Characterization: Size, polydispersity index, zeta potential, *in vitro* release and antibacterial effect using *murine* bone marrow-derived macrophages (mouse strain C57BL/J) were studied and the *in vivo* efficacy was tested in mice.	Incubation with Albumin enhanced the solubility of BTZ. The encapsulation efficiency was 37–60% with more than 50% of the drug released within 4 h. The antibacterial effect of BTZ was enhanced by formulation of the drug inside the NPs.
13 [75]	Validate the high intracellular uptake of nanoparticles	RIF, CUR	Polyethylene sebacate and Tween 80	NP	NPs were prepared by a nanoprecipitation method. Characterization: Entrapment efficiency, particle size, zeta potential, morphology, *in vitro* release, cell viability, and TB efficacy.	NPs with a diameter of 434 nm and zeta potential of −26.89 mV were obtained. The NPs were not toxic and macrophage uptake was enhanced by 1.5×. Importantly, dual-loaded TB drugs could be a promising approach to TB treatment.
14 [76]	Explore the bactericidal effects of selenium nanoparticles	INH,	Selenium and chitosan	NPs	A versatile method was used to prepare the NPs.	The NPs preferentially entered macrophages and accumulated in lysosomes.
15 [77]	Decrease the dose of MOX and AMI	MOX, AMI	PLGA, Alginate	NPs	Two formulations were used: 1- alginate was utilized as a coating polymer of the PLGA NPs. 2- alginate was entrapped in the internal phase to increase the drug encapsulation.	The 1-alginate NPs had a size of 640 nm and negative zeta potential and the 2-alginate particles had a size of 420 nm and positive zeta potential. Both formulations showed a similar drug release profile and were internalized by macrophages. Importantly, dual loading of TB drugs showed an enhanced antibacterial effect compared with single anti-TB drug.
16 [78]	Design of effective and safe drug delivery system	RIF	MPEO-*b*-PCL	NP	NPs were prepared by a nanoprecipitation method.Characterization: Size, Zeta potential, morphology, cytotoxicity, and efficacy on BEAS-2B, J774A.1, and MH-S cell lines.	Formulating RIF within a biodegradable copolymer enhanced the pharmacokinetics and pharmacodynamics of the drug. It was well tolerated, and the antibacterial effects was improved compared with the free drug.
17 [79]	Find better- sustained treatment for TB	RIF, INH	Glyceryl distearate (GDS), precirol ATO- 5, Soybean phospholipids, Tween- 80, Poloxamer-188 and dimethyl sulphoxide (DMSO)	Nanostr uctured carriers and solid nanopart icles	NPs were prepared by mixing in heated aqueous and lipid phases to form emulsion following by homogenization with cold aqueous phase. squalene was added to prepare nanostructured carriers. Characterization: Size, zeta potential, encapsulation efficiency, morphology, *in vitro* drug release, toxicity to THP-1, and	Spherical NPs with a diameter of ∼ 150 nm were prepared and showed a sustained release pattern for INH and RIF over 2 and 7 days, respectively. The NPs were localized in the endosomes and lysosomes. The pharmacokinetic profile of these drugs formulated inside the nanostructured lipid carriers was improved compared with the solid NPs.
					pharmacokinetics in rats when INH or RIF solution was administered orally	
18 [80]	Prepared curdlan nanoparticles loaded with TB drugs	RIF, LVX	Curdlan	NP	NPs were prepared by a nanoprecipitation method.Characterization: Drug loading, *in vitro* drug release, minimum inhibition concentration for *M. smegmatis*, and uptake by RAW 264.7 macrophages.	NPs with a median size of 600 nm sacrificed 95% of bacteria within 4 h, were non-toxic for cells, their uptake by macrophages increased significantly and the drugs were released completely within 70 h.
19 [81]	Using Chitosan to design a mucoadhesive drug delivery to the lungs	RIF	Cetyl palmitate, Tween® 80, and chitosan®	NP	NPs were prepared by a hot ultrasonication method. Characterization: Size, morphology, zeta potential, encapsulation efficiency, stability, *in vitro* release, toxicity on A549 cells.	NPs with a diameter of 245–344 nm and zeta potential of −30 mV were coated with chitosan. The encapsulation of the drug efficiency was 90%. Results showed higher mucoadhesive and permeability to alveolar epithelial cells compared with the uncoated nanoparticles containing the drug.
20 [82]	Design a selective NP drug delivery system.	RFB	Precirol® ATO 5, miglyol-812, stearyl amine, D- (+)- Mannose	NPs	NPs were prepared by high-shear homogenization and ultrasonication. Characterization: Size, zeta potential, morphology, entrapment efficiency, cytotoxicity on human airway epithelial cell line Calu-3, human bronchial epithelial cell line A529, and *murine* macrophage RAW 264.7 cells and drug release kinetics.	Spherical NPs with a size 175–213 nm were coated by mannose, had an encapsulation efficiency of 80% and were stable for 6 months in room temperature. The drug release was faster in acidic pH with a full release of the drug within 25 h. The system showed the possibility of increasing the dose above 100 µg/ml and less than 1000 µg/ml before reaching the inhibitory concentration IC50 which was 238.9, 185.7, and 108.7 μg ml^−1^ for Calu-3, A549s, and RAW macrophages.
21 [83]	Enhance the efficacy and the uptake of RIF	RIF	Stearic and oleic acid as lipid phase, Tween 80, and Phospholipid 80H as surfactants. *N, N-* dimethylformamide(DMF) and methanol as solvent	NPs	Tuftsin-modified peptide (pTUF-OA) was synthesized using a solid-phase approach and NPs were prepared by a microemulsion technique.Characterization: Size, zeta potential, morphology, stability, encapsulation efficiency, *in vitro* drug release, uptake by *Murine* macrophages using a J774 A.1 cell line, and cytotoxicity.	The synthesis of pTUF-OA was successful, and RIF was incorporated in the nanocarriers leading to a nontoxic controlled- release therapy. The NPs with the peptide showed increased uptake by macrophages compared with the NPs without the peptide but both showed a significant improvement in antitubercular effect compared with free RIF.

The abbreviations of drugs listed in the table are Rifampicin: RIF; Capuramycin: CM; Isoniazid: INH; Pyrazinamide: PZA; Streptomycin: SM; Moxifloxacin: MX; Ethionamide: ETH; Linezolid: LZ; Ethambutol: ETM; Thioacetazone: THI; Rifabutin: RFB; Rifapentine: RPN; Benzothiazinone: BTZ; Curcumin: CUR; Moxifloxacin: MOX; Amikacin: AMI; Levofloxacin: LVX; Bedaquiline: BDQ.

## Metal nanoparticles for targeted treatment of TB

The resistance of M.tb to the antitubercular drugs along with a lack of new drugs for the treatment of bacterial infections suggest the need to utilize novel antibacterial agents. In this respect, metal nanoparticles such as silver and zinc have been studied extensively as a potential treatment for many medical conditions [44]. Silver nanoparticles (AgNPs) are antibacterial and have been utilized in several therapeutic [45,46], and diagnostic applications, as well as in optoelectronics [47], and in water disinfection [48]. Studies have reported the antimicrobial effect of AgNPs on resistant strains of bacteria through several mechanisms [49]. These include disturbance of bacterial membranes and cell walls leading to cell leakage by increasing membrane permeability [50], initiating lipid peroxidation and reduction in the levels of the antioxidant, glutathione, depolarization of mitochondria, and oxidative damage of DNA with apoptotic cell death [51], damaging of bacterial cell DNA by binding to its sulfur and phosphorus groups [52], and by releasing Ag ions which play an important antibacterial role by interacting with bacterial cell membranes [53]. Silver nanoparticles in colloidal form and suspension of silver ions with silver nanoparticles in an aqueous medium show superior antibacterial effects by serving as a catalyst which disrupts essential enzymes that microbes need for oxygen metabolism [54]. Similarly, zinc nanoparticles show efficient antibacterial and UV-blocking properties. These attributes are why they are widely used in personal care products, such as cosmetics and sunscreen [55]. Zinc oxide nanoparticles are delivered in a spray form to relieve redness and itching sensations triggered by skin conditions such as lichen planus, eczema, seborrheic dermatitis, psoriasis, and increased skin dryness [56]. Currently, a combination of silver and zinc oxide nanoparticle sprays are prescribed as an antibacterial spray for acute relief of conjunctivitis, skin inflammation, sinusitis, and earache [57]. Many recent studies have been exploring the use of metal nanoparticles for treating TB and MDR-TB as shown in Table 2.

**Table 2:** List of research studies published since 2015 that utilized metal NP or TB drug-loaded metal NP to cure TB

#	Aim	TB drug	Metal agent	Dosage form	Method	Results
1 [84]	Investigate the efficacy of gallium NPs against Human immunodeficiency and tuberculosis coinfection	N/A	Gallium	NP	NP were prepared using a high- pressure homogenizer. Characterization: Morphology, cytotoxicity on THP-1 cells, and growth inhibition against M. tuberculosis (H37Ra) or HIV-1.	Rod-shaped NPs with no toxicity on cells and Ga was released within 15 days. Significant inhibition in growth of TB bacteria was observed with the Ga NP compared with the free metal.
2 [85]	Investigate the efficacy of gallium NP on M. tuberculosis-infected macrophages	N/A	Gallium	NP	NP were prepared using a high- pressure homogenizer. Characterization: Morphology, cytotoxicity on THP-1 cells, and growth inhibition against M. tuberculosis (H37Ra) or HIV-1.	Increased levels of IL-6, IL-8, IL-1β, IL-4, and TNF-α were observed when TB bacteria infected macrophages. Gallium NPs were able to regulate these levels. These NPs inhibited the growth of TB bacteria for 15 days.
3 [86]	Targeting macrophages by gallium NPs	RIF used as control	Gallium	NP	NP were prepared using high- pressure homogenizer. Characterization: Morphology, cytotoxicity and drug uptake by THP-1 cells, growth inhibition against M. tuberculosis (H37Ra), and loading on monocyte-derived macrophages.	The size and zeta potential of NP were dependent on polymer type. The morphology of gallium was approximately rectangular. Gallium NP prepared by dendrimers showed faster uptake by THP-1 macrophages. All formulations showed TB bacteria growth inhibition for 15 days.
4 [59]	Design therapeutic nanoparticles for lung delivery	INH	Iron	NP	INH, D-Leucine NPs (LC NP) and INH PLGA NPs were prepared by spray drying. Characterization: Drug quantification, aerodynamic characterization, size, morphology, *in vitro* drug release, cytotoxicity, and uptake by RAW 246.9.	Irregularly shaped particles with an average diameter of 11 µm with a near-uniform size distribution. Higher INH release was observed from the LC NPs compared with PLGA NPs within 10 h. No cytotoxicity effects were shown with the lowest concentration (10–25 µg/ml) and the highest concentration (500 µg/ml) decreased the cell viability by 52% compared with the control cells.
5 [87]	Design theragnostic nanoparticles encapsulating INH	INH	Iron	NP	Fe-MIL-101-NH_2_ was synthesized by milling in an agate ball mill for 24 h and INH was incorporated by mixing. Characterization: Size, morphology, drug release, and *in vitro* cytotoxicity on fibroblasts L929.	12% of INH was loaded in the NPs with a diameter range of 3.37–6.45 μm based on the micronization method used and a sustained release profile of INH. NPs accumulated inside the L929 fibroblasts with no signs of toxicity.
6 [88]	Explore the antitubercular effect of MgO and ZnO NPs	N/A	MgO, ZnO	NP	MgO and ZnO NPs were prepared. Characterization: Antitubercular activity against MDR and XDR, cytotoxicity on Vero and HepG2 cells.	The NPs didn't show cytotoxicity. The inhibitory effects could be associated with the ZnO NPs. These NPs sacrificed MDR and had a synergetic effect to clear resistant strains.
7 [89]	Characterize silver NPs synthesized by a Streptomyces sp. NH28 strain	N/A	Silver	NP	Silver NP were biosynthesized by an Actinobacterial strain. Characterization: Validation of biosynthesis, determination of functioning groups, Morphology, zeta potential, size, Minimal inhibitory concentration (MIC) of silver NPs against many bacterial strains.	Spherical NPs with a mean size of 19.9 nm with a negative zeta potential of −13.8 mV. The antibacterial effect was observed against all strains and didn't show any toxic effects when exposed to L929 fibroblasts.
8 [90]	Assess the efficacy of silver NPs on TB bacteria	N/A	Silver	NP	NPs were prepared using a single dispersion and stabilized by polyvinylpyrrolidone. Characterization: Size, *in vitro* antitubercular effects against H37Rv strain, and *in vivo* efficacy using mice.	NP with size 43.6 ± 10.7 nm were prepared to enhance the suppression of TB bacteria by 2 fold and decrease the count of TB bacteria in the spleen and lungs by 2 x in mice.
9 [91]	Design a green synthesis of silver NPs	N/A	Silver	Nano composi te	NPs were prepared by a one-step reaction using Chitosan. Characterization: Size, antitubercular effect, and cytotoxicity.	Spherical nanocomposites with a size 11–17.5 nm and IC50 against normal lung cells was 357.2 μg/ml. M. tuberculosis was inhibited by an MIC of 1.95 μg/ml.
10 [92]	Prepare silver NP loaded with antibacterial drugs	Vancom ycin	Silver	NP	NPs were prepared by a citrate reduction process. Characterization: Size, morphology, antibacterial effect against *M. smegmatis.*	Spherical NP with a size 30 ± 3 nm were prepared successfully. The internalization of the drug inside the bacteria was enhanced through formulation with NPs.
11 [93]	Investigate the antimycobacterial activity of silver	N/A	Silver	NP	Silver NP were prepared by a chemical reduction method. Characterization: Size, morphology, MIC against M. tuberculosis H37Rv.	Spherical and tetrahedral silver NP with an average size of 59 nm were prepared. The system showed an antibacterial effect on this TB strain with an MIC of 1 µg/ml.
12 [63]	Fabricate a nanoscale multi-drug delivery system	RIF, PZA	Silver	NP	The biodegradable polymer chitosan–grafted- (cetyl alcohol- maleic anhydride-pyrazinamide) was made by multiple reactions. Silver NP and RIF were incorporated. Characterization: Size, morphology, drug release, *in vitro* cytotoxicity on Vero and THP-1 cells, and antitubercular activity.	FTIR confirmed the successful synthesis of silver, RIF, and polymer containing PZA. PZA and RIF were in an amorphous phase with a size of 140 nm and their full release was performed within 12 h. The combination therapy showed better antitubercular effect than their single administration.
13 [94]	Design and characterize biodegradable silver NPs	N/A	Silver acetate, silver carbene complexes (SCCs)	NP	A solution of silver was added to the Diblock Copolymer, Poly (butynyl phosphotriester)210-block- Poly(L-lactide)50 (PPE210-b- PLLA50). Characterization: Stability, release kinetics, and antibacterial activity *Staphylococcus aureus* and *Escherichia coli.*	50% of silver was released within 2.5–5.5 h. Encapsulating silver within NPs increased the minimum inhibition order by 70%.
14 [95]	Green synthesis of silver NPs by yeast	N/A	Silver chloride	NP	NPs were prepared using yeast. Characterization: Crystalline nature of NP, size, morphology, antitubercular activity against M. tuberculosis H37Rv.	Spherical NPs with a diameter of 17 nm sacrificed the bacteria by inducing oxidative stress. These NPs showed a 95% reduction in TB bacteria with an administrated dose of 37 µg/ml.
15 [96]	Investigate the changes in immune response to TB when silver NP is administered	N/A	Silver, carbon black	NP	Different concentrations of silver NP with different stabilizers were used in the preparation method. Characterization: Size, morphology, cell viability on human monocyte-derived macrophages (MDM) and *in vitro* release.	Different formulations showed different sizes and zeta potentials. Importantly, silver NPs reduced cellular viability, increased IL8, and decreased IL10 mRNA expression when exposed to MDM. For the TB- infected MDM, silver NP suppressed. *M. tb*-induced expression of IL1β, IL10, and TNFα mRNA, and TB bacteria was inhibited by silver NPs.
16 [97]	Evaluate the biological risks associated with exposure to NP	N/A	Silver, black carbon	NP	Silver NPs were prepared by bath reduction using sodium citrate. Characterization: Cell viability on human monocyte-derived macrophages (MDM).	After 4 h of silver NP exposure to MDM, no toxicity was observed. But after 24 h, the cell viability was reduced by 60–70%. Silver NPs up-regulated Hsp72 leading to suppress NF-kB induced by *M.tb*, thus hosting immune responses.
17 [98]g	Evaluate the antitubercular activity of metal NPs	N/A	Silver, Gold	NP	NPs were synthesized from plants, such as Barleria prionitis, Plumbago zeylanica, and Syzygium cumini. Characterization: *In vitro* and *ex vivo* minimum inhibitory concentration, internalization by macrophage, and cytotoxicity.	A combination of gold and silver NP had the most striking antitubercular effect with a MIC of less than 2.56 µg/ml. No antibacterial effect was observed for gold NPs with concentrations of < 100 µg/ml. Metal NPs entered macrophage cells.
18 [99]	Compare the efficacy of silver and gold NPs	N/A	Silver, Gold	NP	Biological synthesis of silver and gold NPs was done using environmental bacterium. Chemical synthesis was performed using reduction reactions. Characterization: Size, Morphology, antitubercular effects *in vitro* and ex vivo using infected THP-1 cells and cytotoxicity assays.	Ag and AuNPs were spherical and Polyhedral in morphology, respectively. AgNPs showed better antitubercular efficacy than the gold NPs.
19 [98]	To investigate the efficacy of phytogenic metal nanoparticles	N/A	Silver, gold	NP	NPs were prepared using medicinal plants, such as Barleria prionitis, Plumbago zeylanica, and Syzygium cumini. Characterization: Size, morphology, MIC against H37Rv, toxicity, and uptake by THP-1 macrophages.	The size and morphology were dependent on the phytogenic source. Mixed Ag and Au spherical NPs prepared by *Syzygium cumini* with a diameter of 10–20 nm showed the most powerful formulation against TB bacteria. 45% cell viability was observed at a dose of 30 µg/ml of the mixture of Au-Ag NPs. Silver NPs showed more potent antibacterial effects than gold NPs.
20 [100]	Synthesized NPs using environmental bacteria	N/A	Silver, zinc	NP	Silver and zinc oxide NP were prepared using a bacterial culture of *Pseudomonas hibiscicola*. Characterization: Size, morphology, cytotoxicity on a Vero cell line, and testing of the antimicrobial efficacy against many strains including Mycobacterium tuberculosis H37Rv.	Polydisperse spherical silver and zinc NPs with a mean size of 39 and 62 nm were prepared. IC50 values for silver and zinc NPs were 5.54 and 6.24 mg/ml. Authors concluded that metal NPs could enhance the antibacterial efficacy of many drugs like gentamicin.
21 [101]	Evaluate the antitubercular effects of mixed metal oxides	N/A	Silver, ZnO	NP	NPs were prepared by chemical reduction. Characterization: Size, morphology, toxicity on THP-1 cells, and antitubercular effects against M. tb.	Spherical NPs with a diameter of 30–80 nm. MIC ratio of 8ZnO:2Ag NPs against *M. tb* was detected at ratio of ∼1/32 of the initial concentration of Ag NPs and ZnO NPs were estimated at ∼20 ppm and ∼60 ppm. Silver did not show antitubercular effects at any of the applied doses, while ZnO NPs showed a potent antibacterial activity at ∼1/128 and toxic effects on the cells.
22 [102]	Determine the effective ratio of mixed metal NPs	N/A	Silver, ZnO	NP	NP were prepared by bath reduction in sodium citrate. Characterization: size, shape, cytotoxicity on THP-1 cells and antibacterial efficacy using Mycobacterium tuberculosis (H37RvMTB).	Spherical particles with a size of 13 nm for Ag NPs and 4 nm for ZnO NPs. 0.663 ppm of 5Ag:5ZnO showed effective antibacterial results with no toxicity to THP-1 cells. The combination of both metals together showed better results.
23 [103]	To design biodegradable microparticles containing mixed metal NPs to be delivered to the lungs	RIF	Silver, ZnO	MP	Capped silver NP were prepared by a bath reduction method and capped ZnO NP were prepared using an organometallic route. The MPs were prepared using a solvent evaporation method. Characterization: Size, morphology, elemental composition, antitubercular effect against H37Ra *M. tb*, and cytotoxicity on THP-1 cells.	ZnO and silver NPs were formulated within PLGA microparticles with a diameter of 4 µm. Selective uptake of the MPs by *M.tb* infected macrophages and zinc and silver ions were released which disrupted the *M.tb* cell wall. This formulation increased the potency of RIF by 75%.
24 [60]	Investigate the antibacterial effect of zinc and silver	N/A	Silver, ZnO	NP	Ag and ZnO NPs were prepared by a chemical reduction method. Characterization: size, morphology, MIC against M. tuberculosis H37Rv, MDR, and XDR.	Spherical silver and ZnO NPs with a size of 5.4 ± 2.6 nm and 9.3 ± 3.9 nm were produced. The MIC of all of the formulations was 1 µg/ml. Silver and zinc NP showed a bacteriostatic effect against MDR and XDR strains of *M.tb.*
25 [104]	Explore the antibacterial effect of TiO_2_ NPs	N/A	TiO2	NP	NP were prepared by a sol-gel method using TiOSO_4_. Characterization: Size, morphology, antitubercular effect on TB bacteria, and cytotoxicity using lung bronchus cells.	Spherical NP with diameter of 16 nm showed size and concentration-dependent antitubercular effects with a 3–4 times decrease in TB metabolic activity with very minimal toxicity when the maximum dose was applied.
26 [105]	Green synthesis of NPs	N/A	ZnO	NP	NP were prepared using the extracted *Limonia acidissima L.* and a zinc nitrate solution. Characterization: Particle size and distribution, anti-TB activity against H37 RV strain, and morphology.	Monodisperse spherical NPs with a diameter of 12–53 nm were prepared. The NPs inhibited the growth of TB at a concentration of 12.5 µg/ml.
27 [106]	Green synthesis of ZnO	N/A	ZnO	NP	A hydrothermal combustion method was used for Phyto- synthesis ZnO-nanoparticles from Canthium dicoccum. Characterization: Size, zeta, potential, crystallinity structure, antibacterial activity and MIC	Rod-shaped ZnO particles with an average size of 33 nm. *B. subtilis* inhibited bacteria with a MIC value of 78.12 µg/ml. TB growth was inhibited by 25–100 µg/ml of ZnO nanorods.
					against positive and negative-gram bacteria and antitubercular efficacy against Mycobacterium tuberculosis (ATCC No- 27294).	
28 [107]	Green synthesis of ZnO as antitubercular agent	N/A	ZnO	NP	ZnO NPs were prepared by Capparis zeylanica. Characterization: Size, Morphology, Antitubercular effect on TB bacteria.	Spherical NPs with a diameter of 34 nm. The maximum diameter of inhibition zone was observed in at (100 μg/ml) against *M. tuberculosis* (35 ± 1.86).
29 [108]	Explore the synergistic effect of ZnO and RIF NPs.	RIF	ZnO	NP	ZnO NP were prepared by precipitation in liquid. Characterization: Size, morphology, antitubercular activity against Wild-type (WT) *Mycobacterium smegmatis* mc2155, zeta potential, and MIC.	Uniformly distributed ZnO NPs with a diameter of 11 nm and zeta potential of +19.1 mV. The MIC for the ZnO NP was 256 µg/ml but 32 µg/ml of ZnO NPs were able to reduce the MIC of RIF from 64 µg/ml to 16 µg/ml which confirms a synergistic effects between both antitubercular agents.

Table 2 shows the antitubercular effect of metal/metal oxide NPs, and TB drugs loaded with metal/metal oxide NPs, against different strains of M.tb bacteria. Importantly, all metal-containing agents, such as silver, gold, titanium, zinc oxide, and gallium, showed a bactericidal effect when applied to M.tb [58]. Similarly, a synergistic effect was reported for first-line TB drugs such as Rifampicin when formulated within a metal/metal oxide NP system leading to an increase in the antitubercular effect of these drugs and reducing their minimal inhibitory concentration [59]. Interestingly, silver and zinc oxide NPs showed only a bacteriostatic effect when used against resistant strains of M.tb [60]. These metal/metal oxide NPs showed low toxicity when incubated with eukaryotic THP-1 and Vero cells, suggesting the possibility of using an advanced therapy containing a cocktail of metal/metal oxide NPs along with TB drugs to improve the efficacy of TB treatment and shorten the duration of therapy. Similarly, metal NPs showed a significant improvement in the diagnostic approach to TB. Employing gold NPs in the detection of TB DNA for diagnostic purposes, using a paper-based analytical platform, resulted in a highly sensitive detection limit of 1.95 × 10^–2^ ng/ml for TB DNA [61].

## Conclusions

TB remains a significant global disease that causes high morbidity and mortality for which current therapeutic strategies are often inadequate; consequently, it continues to be a major health concern. New strategies are being examined, broadening the scope of the research, with innovative novel approaches to improve treatment, and reduce mortality. Currently, the number of newly formulated TB drugs is low. Different technologies such as micro- and nano- technologies are being used as carriers for targeted M.tb treatment by encapsulating single and combinations of traditional antibiotics, as well as combining antibiotics with antibacterial metals; the metal nanoparticles have an additive/synergistic effect allowing use of lower doses of drug(s) with reduced side effects, whilst exhibiting high efficacy. Promising results report increasing the antitubercular efficiency of many first and second-line TB drugs when formulated within nano- and microparticles which facilitates drug uptake by M.tb. infected macrophages [62]. Similarly, utilizing metal/metal oxide NPs together with TB drug(s) increased their efficacy [63].

Importantly, there is a need to establish reliable *in vivo* models to examine the impact of the metal NP- drug formulations in animal models as the number of *in vivo* studies on these drug formulations is very limited. Metal-based formulations have an element of risk related to the potential toxicity of the metal, the dose of which would need to be carefully monitored and adjusted for human use.

Organic and metal micro- and nano-carriers offer great potential for more effective delivery of TB drugs to the affected site, alone and in combination, to increase their potency, particularly in the presence of antibacterial metal nanoparticles. Introduction of potent, novel, and repurposed drugs will increase the impact of such systems and hopefully reduce the impact of M.tb. on human health.

## Summary

- The Tuberculosis epidemic continues, claiming 1.5 million lives a year and causing ∼10 million new case diagnosed annually.
- Antimicrobial treatment is available but takes too long and has side effects leading to poor patient compliance and the emergence of drug resistant organisms.
- The time has now come to make use of the latest in drug formulation and delivery science to develop better ways to administer antimicrobial treatment to TB patients in such a way as to maximize efficacy and minimize treatment time and side effects.
- These improved delivery formulations would make best use of the currently available drugs, as well as those in the pipeline.
- Micro- and nano-particle technologies are now sufficiently mature for use in robust inhalation drug delivery systems to deliver antimicrobial therapy more effectively to the major site of Tuberculosis in the lung.

## Abbreviations

BCG: Bacillus Calmette-Guérin
DOTS: Directly Observed Therapy Short-course
INH: Isoniazid
LTBI: latent stage tuberculosis
MDR: multi-drug resistant
MPs: microparticles
NPs: nanoparticles
TB: tuberculosis
